# Evolution of *Acinetobacter baumannii In Vivo*: International Clone II, More Resistance to Ceftazidime, Mutation in *ptk*

**DOI:** 10.3389/fmicb.2017.01256

**Published:** 2017-07-10

**Authors:** Xiaoting Hua, Zhihui Zhou, Qing Yang, Qiucheng Shi, Qingye Xu, Jianfeng Wang, Keren Shi, Feng Zhao, Long Sun, Zhi Ruan, Yan Jiang, Yunsong Yu

**Affiliations:** ^1^Department of Infectious Diseases, Sir Run Run Shaw Hospital, College of Medicine, Zhejiang UniversityHangzhou, China; ^2^Key Laboratory of Microbial Technology and Bioinformatics of Zhejiang ProvinceHangzhou, China; ^3^State Key Laboratory for Diagnosis and Treatment of Infectious diseases, Collaborative Innovation Center for Diagnosis and Treatment of Infectious Diseases, The First Affiliated Hospital, College of Medicine, Zhejiang UniversityHangzhou, China; ^4^Department of Respiratory Diseases, The Affiliated Hospital of Hangzhou Normal UniversityHangzhou, China; ^5^Department of Clinical Laboratory, Sir Run Run Shaw Hospital, School of Medicine, Zhejiang UniversityHangzhou, China; ^6^Department of Clinical Laboratory, Hangzhou General Hospital of Zhejiang Provincial Corps, Chinese People’s Armed Police ForcesHangzhou, China

**Keywords:** *Acinetobacter baumannii*, within-host evolution, whole-genome sequencing, mucoid, ICL-II

## Abstract

*Acinetobacter baumannii* is an important nosocomial pathogen worldwide. A more comprehensive understanding of the within-host genomic evolution of *A. baumannii* would provide a molecule basis for improving treatment of *A. baumannii* infection. To understand the evolutionary mechanism facilitating *A. baumannii* survived in human body, we here reported the genomic analysis of *A. baumannii* isolated sampled from Chinese patients. We used whole-genome sequence of *A. baumannii* isolates from the same patient to determine single-nucleotide variants, insertion sequence mapping, and gene change. The MICs for 10 antimicrobial agents were determined. Motility assay and microscopy were performed on the isolated pairs harboring *ptk* mutations. The gene *ptk* encoded a putative protein tyrosine kinase involved in the production of capsular polysaccharide. Approximately half (39/86) of the strains isolated from the same patient harbored the same MLST patterns, and during the replacement of international clonal lineage II (ICL-II) and non-ICL-II strains, most of the alteration was that non-ICL-II strain was replaced by ICL-II strain (10/12). *A. baumannii* was resistant to major antimicrobial agents, whereas the strains were more resistant to ceftazidime, azithromycin, and sulfonamides after within-host evolution. Isolates from the ICL-II lineage displayed greater resistance to antimicrobial agents than non-ICL-II isolates. Isolates from ICL-II harbored more resistance genes and mobile elements than non-ICL-II strains. Several lineages evolved a more mucoid phenotype. Genome sequencing revealed that the phenotype was achieved by genetic changes in the *ptk* gene. ICL-II (especially ST195 and ST208) was the terminal destination for bacteria after within-host evolution. These results indicate that the molecular basis and the treatment for ICL-II strains needed further investigation.

## Introduction

It is important to achieve comprehensive understanding of how bacteria evolve during human host infections to be able to treat infections effectively. Recent advances in DNA sequencing technologies have made it possible to identify genetic changes between the genome of the same strains over shorter or longer periods of time to reveal important adaptations to the host environment (Wilson, 2012). The comparison of bacterial pathogens’ genome over time from individual patients during infection and therapy will advance the understanding of the bacteria’s evolution, epidemiology, and antibiotic resistance (Marvig et al., 2013). It could determine the relationship among infecting bacteria isolates and gain insight in genetic adaptation in infected host and response to antibiotic treatment. Several studies have focused on the genomic evolution of pathogens causing acute infections, such as *Yersinia pestis* (Morelli et al., 2010), *Neisseria meningitidis* (Klughammer et al., 2017) and *Vibrio cholera* (Mutreja et al., 2011), or causing long-term infections, such as *Pseudomonas aeruginosa* (Marvig et al., 2013) and *Acinetobacter baumannii* (Wright et al., 2016).

*Acinetobacter baumannii* has a greater correlation with resistance to antibiotics and higher mortality among bacteremic patients, than does *A. nosocomialis* (Chuang et al., 2011). International clonal lineage II (ICL-II) represents the largest and most geographically diverse CC (Zarrilli et al., 2013; Kamolvit et al., 2015; Chen et al., 2017). High-virulence, healthcare-associated *A. baumannii* were also isolated, which altered the general consideration that *A. baumannii* was a low-virulence organism (Jones et al., 2015). Many virulence mechanisms have been identified in *A. baumannii*, including capsule formation and siderophore-mediated iron acquisition systems (Russo et al., 2010; Cerqueira and Peleg, 2011). Previous genomic analysis of *A. baumannii* isolates from the same patient over time showed the enrichment in mutation associated with antibiotic and host response (Wright et al., 2016).

To understand the evolutionary mechanism facilitating *A. baumannii* survived in human body, we here reported the genomic analysis of *A. baumannii* isolated sampled from Chinese patients. In total, we sequenced the genomes of 172 *A. baumannii* isolates sampled from 86 different patients between 2011 and 2015. Genomic analyses and MIC assays were performed to provide a comprehensive view of within-host evolution in *A. baumannii*.

## Materials and Methods

### Bacterial Strains and Genome Sequencing

This study encompassed 172 isolates of the *A. baumannii* strains that were sampled from 86 patients in three hospitals in Hangzhou, China in a pair-wise longitudinal manner. The criterion of isolate selection was listed followed: (1) The bacteria were isolated from the same patient; (2) The time interval between two isolates is longer than 1 month. The isolation and identification of *A. baumannii* were performed as previously described (Ruan et al., 2013). All 172 isolates were sequenced at Zhejiang Tianke (Hangzhou, China) on an Illumina HiSeq2000 platform (Illumina, San Diego, CA, United States). In general, more than 300-fold coverage was obtained for each genome sequences. Sequence data from all isolates were deposited in GenBank under the accession numbers indicated in the Supplementary Table S1.

### Ethics Statement

This study was approved by each ethics committee of Sir Run Run Shaw Hospital College of Medicine Zhejiang University, The First Affiliated Hospital College of Medicine Zhejiang University and Hospital of Zhejiang Corps, Chinese People’s Armed Police Forces. Written informed consent was waived by all Committees. The need for a written consent by the Ethical Committees was waived and this was approved by all committees, due to the fact that only the bacterial isolates were taken from the patients and the study with confidentially fully guaranteed.

### Antimicrobial Susceptibility Testing

Antimicrobial susceptibility testing was performed in Muller Hinton (MH) broth using the two-fold serial agar dilution method according to the guidelines of the Clinical and Laboratory Standards Institute (CLSI) (Patel et al., 2015). The following antimicrobial agents were tested: amikacin (AK), ciprofloxacin (CIP), chloramphenicol (C), streptomycin (SM), sulfonamides (S), meropenem (MEM), tetracycline (TET), ceftazidime (CAZ), azithromycin (AZM), and kanamycin (KANA). The concentration of the sulfonamides ranged from 1.25 to 320 mg/L, and the concentration of the other antimicrobial agents ranged from 0.125 to 256 mg/L. The Tigecycline MIC was determined by using the CLSI broth dilution method, and *E. coli* ATCC 25922 was used as Quality control strain. Shapiro–Wilk normality test was used to detect whether the data is parametric. Statistical analysis was performed by using pairwise student *t*-test for parametric variables and Mann–Whitney *U* test for non-parametric variables.

### Mutation Detection and Analysis

Sequence types (STs) and resistance genes were derived directly from short reads via SRST2^1^ using both the Oxford scheme and the Institute Pasteur scheme (Inouye et al., 2014). To detect the presence of transposon carrying *bla*_OXA-23_, the genome sequences of all the strains was blasted with the sequences of Tn2006, Tn2008, and Tn2009. The minimum spanning tree was calculated by Prim’s algorithm using the MLST plugin in the BioNumerics 6.6.4 software (Applied Maths, Sint-Martens-Latem, Belgium) incorporated with the eBURST algorithm. Clonal complexes (CCs) were defined as containing at least three STs sharing the same allele numbers in at least six of seven loci. The number and position of insertion sequence were identified by ISseeker via annotating IS elements in draft genome assemblies with the reference genome (XH386, ST208) (Adams et al., 2016). XH386 was belonged to ICL-II isolate and isolated from China in 2014, and was selected as a reference genome for the majority of isolates in this study are ICL-II.

For the comparison between the strain pairs with the same STs, the sequence reads of the former isolate were *de novo* assembled using CLC Genomic Workbench 8.5.1 (Qiagen, Aarhus, Denmark). The assembled contigs were annotated with Prokka 1.11 (Seemann, 2014). The copy number of resistance genes were detected by CNV-seq via mapping the reads to the sequence of resistance genes (Xie and Tammi, 2009). The identification of transposon harbored *bla*_OXA-23_ was based on blast the genome sequences of the sequenced strains with the sequence of Tn2006, Tn2008, and Tn2009. The sequence read mappings of the latter isolate against annotated contigs and mutation prediction were all performed with Breseq (Deatherage and Barrick, 2014). The pan genome analysis was performed using Roary (Page et al., 2015), and the core genes were identified. The genes harboring mutations were mapped to the core genes to obtain the mutant gene frequency.

### Microscopy

Bacterial capsules were detected using the India ink method (Geisinger and Isberg, 2015). Briefly, three bacterial colonies were mixed with 1 ml of 0.9% NaCl solution, and India ink was added. Finally, the bacteria were analyzed by light microscopy (1000×).

### Motility Assay

The motility of all isolates was tested on MH medium supplemented with 0.3% (wt/vol) agarose as described previously (McQueary et al., 2012). Briefly, the MH plates were inoculated with single colonies in the middle of the plates to measure swarming motility. The measurement was performed after incubation at 37°C for 24 h and the motility zones were measured triplicate. Statistical analysis was performed by using student *t*-test.

## Results

### Clinical Collection of Bacterial Isolates for Sequencing

In this study, we sequenced the whole genomes of 172 isolates of *A. baumannii* sampled from 86 patients in sputum. Two groups of isolates for each patients were chosen for sequencing to gain insight into the evolution of *A. baumannii in vivo*. The first group of isolates was designated as Group A, and the second group was designated as Group B. The average time span between obtaining the *A. baumannii* isolates from each patients was 46 days (range = 31–394 days) (**Supplementary Figure S1**).

### MLST and Clonal Complexes

The MLST profiles were generated from the WGS data. Using the Pasteur MLST scheme, a total of 12 different STs were identified, including eight existing STs and four novel STs (Supplementary Table S1). During the process of MLST identification, XH551, XH653, XH654, XH762, and XH796 were *A. nosocomialis*, while XH765 was *A. pittii*. ST2 (using the Pasteur scheme) was the predominant ST, comprising 80% of the isolates. Using the Oxford MLST scheme, there were 24 different ST, nine of which were novel. Furthermore, 139 isolates belonging to ST2 (using the Pasteur scheme) were divided into types including ST195, ST208, ST191 using the Oxford MLST scheme. Among them, 59 isolates were ST195, 24 isolates were ST208 and 22 isolates were ST191. A minimum spanning tree analysis indicated that 12 of the 24 STs were clustered into two CCs which were ICL-II and CC462. ST704 (*A. nosocomialis*) and ST642 were two locus variants of ST1343 (*A. nosocomialis*) and ST447, respectively. They were clustered into CC10. And ST1342, ST1344, ST1345, and ST1346 were singletons.

Of the 86 isolate pairs, 39 were the same STs (one isolate pairs were *A. nosocomialis*: XH653 and XH654). In the pairs harboring the same STs, 33 pairs belonged to ICL-II. The isolate pairs were separated into three categories: (i) different STs; (ii) the same STs and belonging to ICL-II; or (iii) the same STs and not belonging to ICL-II (non-ICL-II).

**FIGURE 1:**
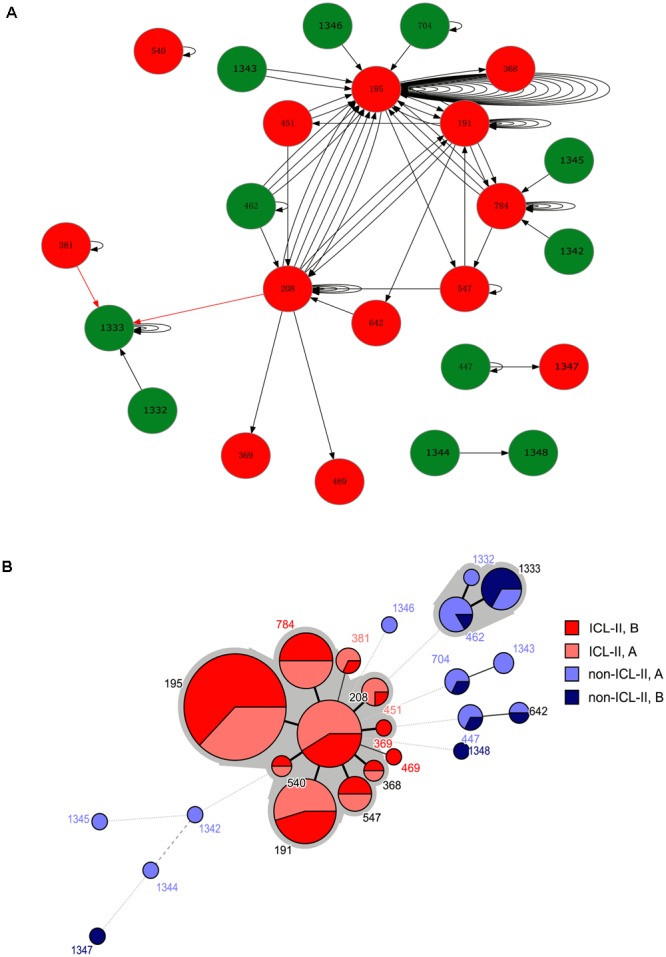
**(A)** A schematic of *A. baumannii* within-host evolution. The arrow indicated the alteration of two isolates in one patient. A circle at the head of the arrow indicates that the first isolates in the patients belonged to Group A. A circle at the end of the arrow indicates that the second isolates in the patients belonged to Group B. The black arrow indicates an alteration between the same STs or from non-ICL-II to ICL-II. The red arrow indicated an alteration from ICL-II to non-ICL-II. The number within the circles is the MLST. The color of the circle indicates whether the isolate belonged to ICL-II: Red: ICL-II; Green: non-ICL-II. **(B)** Minimum spanning tree analysis of all genome-sequenced *A. baumannii* isolates based on MLST data. Each circle represents an independent ST. The size of each circle indicates the number of isolates. The lines connecting the circles indicate the relationship between different STs. Different types of lines represent the difference in one allele (solid line), two alleles (dashed line), and three or more alleles (dotted lines). Two colored zones surrounding the STs indicate that they belong to the same clonal complex.

For the harboring different STs isolate pairs, it was common that the strain from ICL-II was replaced by another strain from ICL-II, and ST195, ST208 and ST191 played an important role in within-host evolution for they represent the STs that were most frequently switched to during within-host evolution (**Figure 1**). During the replacement of ICL-II and non-ICL-II strains, most of the alteration was that non-ICL-II strain was replaced by ICL-II strain (10/12). It might cause by the competition of different types of strains *in vivo*.

### Antibiotic Resistance

**FIGURE 2:**
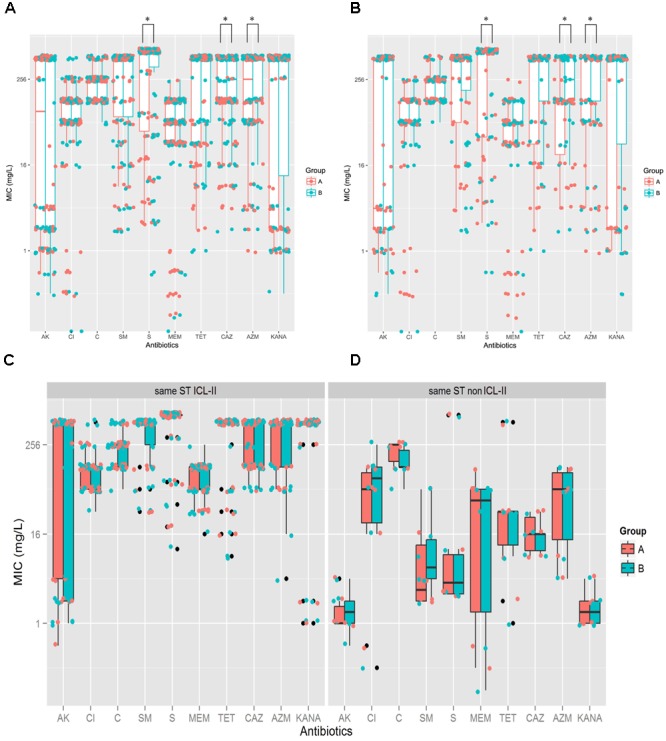
*A. baumannii* exhibited more resistance after within-host evolution, among the isolates, ICL-II strain demonstrated more resistance than non-ICL-II strains did. **(A)**. For total sample, boxplots for Groups A and B showing the mean ratio of MIC for CAZ between Groups A and B. Dots indicate the detailed MIC values ratio for CAZ. **(B)** For the isolate pairs harboring different STs, boxplots for Groups A and B showing the mean ratio of MIC for AK, CAZ, MEM, and AZM between Groups A and B. Dots indicate the detailed MIC values ratio for that antibiotic. **(C)** MIC distributions for 10 antibiotics in whole genome-sequenced *A. baumannii* isolate pairs harboring the same ICL-II STs. Colors indicate the isolate in Groups A and B. Boxplots for each group indicate the mean MICs against the respective antibiotic. Dots indicate the detailed MIC value for that antibiotic. **(D)** MIC distribution for 10 antibiotics in all genome-sequenced *A. baumannii* isolate pairs harboring the same non-ICL-II STs.

To explore whether the evolution of *A. baumannii* would alter the antibiotic resistance profiles, the MICs for 10 antibiotics were determined and compared between Groups A and B by Mann–Whitney *U* tests. For the total sample, Group B demonstrated higher resistance than Group A did for CAZ (*P* = 0.006), AZM (*P* = 0.035) and S (*P* = 0.046) (**Figure 2A**). For the isolate pairs harboring different STs, three antibiotics resistances were significantly increased when comparing Groups A and B, including CAZ (*P* = 0.002), AZM (*P* = 0.011) and S (*P* = 0.026) (**Figure 2B**). No significant differences were observed between isolate pairs harboring the same STs (ICL-II and non-ICL-II) (**Figures 2C,D**). Furthermore, ICL-II isolates exhibited a higher resistance to antibiotics compared to non-ICL-II isolates (**Figures 2C,D**).

**FIGURE 3:**
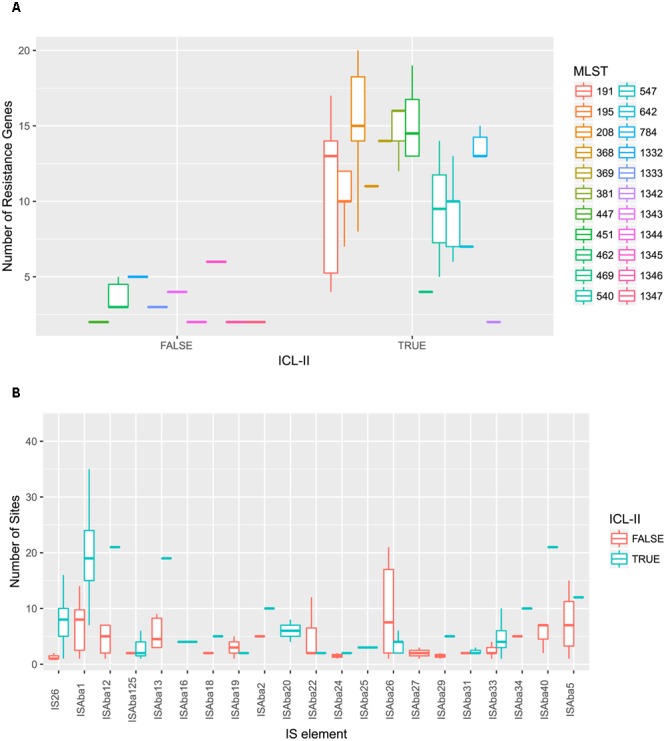
*A. baumannii* ICL-II presented a higher number of resistance genes and mobile element. **(A)** *A. baumannii* ICL-II harbored more resistance gene than non-ICL-II isolates. Colors are indicated for isolates belonging to different MLST. Boxplots for each MLST display the mean number of resistance genes. Dots indicate the number of resistance gene for isolate. **(B)** *A. baumannii* ICL-II harbored more IS*Aba1* than non-ICL-II isolates. Colors are indicated for isolates belonging to different MLST. Boxplots for each MLST display the mean number of mobile element. Dots indicate the number of ISAba1 for each isolate.

**FIGURE 4:**
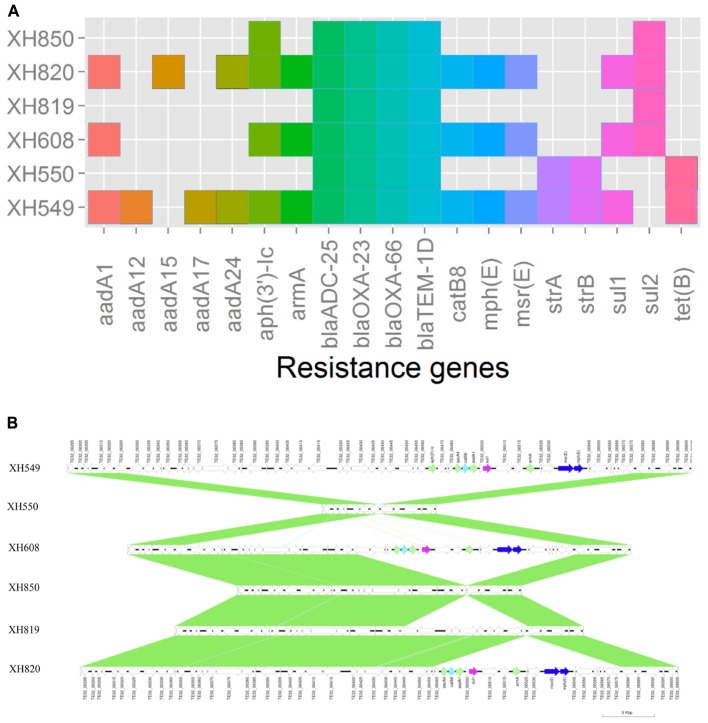
Gene acquire and loss in three isolates pairs of *A. baumannii*. **(A)** Heatmap of resistance genes in three isolates pairs. Both XH549 and XH608 represent resistance gene loss. XH819 was shown to acquire resistance genes. **(B)** Comparative analysis of the genomes of three isolates pairs generated by Easyfig. The arrows for resistance genes are shown in different colors.

**FIGURE 5:**
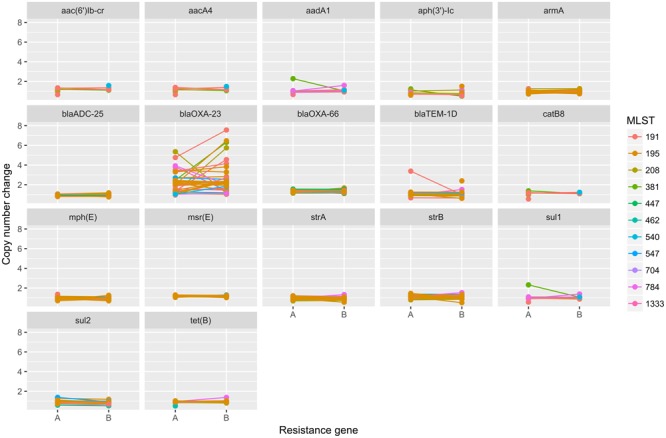
The copy number change of resistance genes in Groups A and B in *A. baumannii*. The copy number of resistance genes were detected by cnv-seq via mapped the WGS data to the sequence of resistance genes.

**FIGURE 6:**
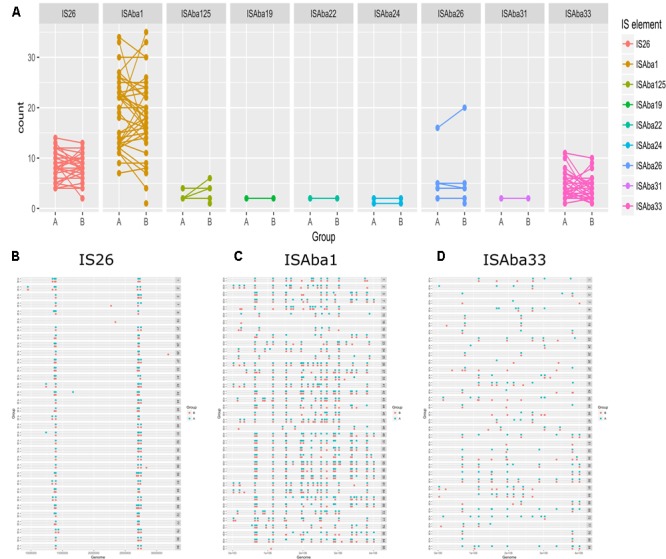
The distribution of IS elements in Groups A and B in *A. baumannii*. **(A)** The copy number of IS elements in Groups A and B. The position of IS26 **(B)**, ISAba1 **(C)**, ISAba33 **(D)** in Group A and Group B.

**FIGURE 7:**
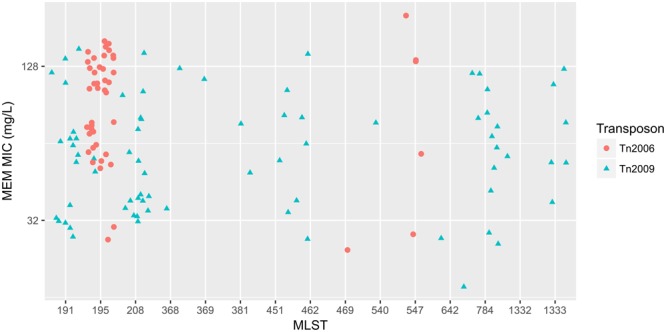
The relationship between meropenem MICs and Transposons carrying *bla*_OXA-23_ in *A. baumannii*. The *x*-axis showed the MLST of strains in *A. baumannii*. The *y*-axis presented meropenem MICs. Red circle: Tn2006; cyan triangle: Tn2009.

To elucidate the relationship between antibiotic resistance and the resistance genes, we detected the distribution of resistance genes among the isolates via SRST2 (**Supplementary Figure S2**). ICL-II isolates contained more resistance genes than non-ICL-II isolates did (**Figure 3A**). To estimate the role of IS elements in mobilizing resistance genes, the number and the position of insertion sequence were identified by ISSeeker. ICL-II isolates harbored higher copy number of *ISAba1*, ISAba33 and IS26 than non-ICL-II isolates did (**Figure 3B**). Combining the data of resistance genes and MICs of *A. baumannii*, resistance gene loss and acquisition were detected in three pairs of isolates (**Figure 4A**). For example, *armA* conferred high-level resistance of AK. The presence and absence of *armA* were correlated with the MICs for AK in the three pairs of isolates (Supplementary Table S1). We identified resistance gene acquisition and loss events during the within-host evolution. After mapping the reads of three pairs of isolates to the reference genome XH386, a 50 kb chromosomal fragment containing multiple resistance genes was absent in XH550 compared to XH549 (**Figure 4B**). Further, a 38 kb chromosomal fragment that included resistance genes was absent in XH850 compared to XH608. Additionally, there was a 18 kb fragment insertion at the same site in XH820 compared to XH819. To investigate whether the copy number of resistance gene changed during within-host evolution, the copy number of resistance gene was detected by CNV-seq in Groups A and B. The result showed that only *bla*_OXA-23_ displayed copy number dynamic during within-host evolution (**Figure 5**). We also detected the copy number or position change in Groups A and B. For copy numbers, IS26, ISAba1, and ISAba33 were the most frequency changed IS element between Groups A and B (**Figure 6A**). For the position of IS elements, the mobilization of IS elements were common observed in within host evolution (**Figures 6B–D**). The genome sequences of all the strains was blasted with Tn2006, Tn2008, and Tn2009. Among all strains, 64 strains harbored Tn2006, and Tn2009 was appeared in 93 strains. For STs, Tn2006 presented in ST195, ST469, and ST547, while Tn2009 showed in all STs except ST469 and ST547 (**Figure 7**).

### SNP Analysis

**FIGURE 8:**
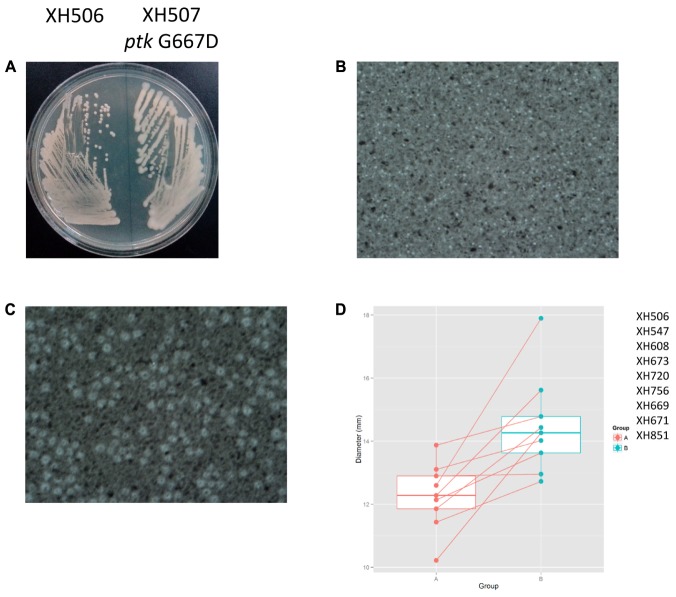
Spontaneous point mutations in *ptk* resulted the misregulation of capsular exopolysaccharide production and higher motility. **(A)** Colony morphology on MH plates. **(B,C)** Capsules of XH506 and XH507 (*ptk* G667D) were analyzed by India ink staining. **(D)** Spontaneous *ptk* mutants showed higher motility than the wild type. The *y*-axis indicates the measurement of diameter (mm) of the cell halos of each isolate pairs harbored *ptk* mutations in Group B. The data are represented as the means ± SD of experiments that were performed in triplicate.

To detect the within-patient diversity of the clonal population, we counted the number of SNPs that each isolate had accumulated based on the first isolate of the pair. The greatest pairwise genome SNP counts from the same patients was less than 20 SNPs (**Supplementary Figure S3A**). We identified a total of 15 genes that were mutated more than one time (**Supplementary Figure S3B**), and the detailed SNPs are shown in **Table 1**. Among them, the *ptk* (also referred as *wzc*) mutation occurred in nine isolate pairs. The gene product of *ptk* has a predicted role in capsule biosynthesis. For example, XH507 generated a mucoviscidose colony phenotype compared with XH506 for it harbored G667D mutation in *ptk* (**Figure 8A**). XH507 also displayed capsules with increased thickness when inspected with India ink (**Figures 8B,C**). Genome sequencing showed that the *ptk* mutations appeared in *ptk* conserved domains (**Supplementary Figure S4**). To investigate whether the genes required for the production of the K1 capsule are required for motility, we performed a motility assay for these isolates. These *ptk* mutants demonstrated higher motility levels compared to those of the parental strain (student *t*-test, *P* = 0.003) (**Figure 8D**). The result suggested that the capsular polysaccharide contributed to the motility phenotype.

**Table 1:** The SNPs identified in *A. baumannii* during infection.

Gene	Gene_product	Freq	Amino acid alteration					
ptk	Tyrosine-protein kinase, autophosphorylates	9	S550L	G667D	H118R	L528F	A531V	L669F	T650A	R483H	I75N
TE32_03770	Type I secretion C-terminal target domain-containing protein	8	T22T	T62R	A36A	I50I	I6I	A52D	A17T	I74I	
TE32_03765	Putative cell-surface adhesin	4	P728P	T720T	V712V	T728T					
TE32_02230	Outer membrane protein A precursor (190 kDa antigen)	3	V469V	L446L	G308G						
tadA	Deaminase	3	Y126C	S37P	P37S						
bauA	Outer membrane receptor	3	A391T	A391T	A391V						
adeS	Two-component sensor	3	N127K	Y31N	R161C						
Hypothetical protein_1	Hypothetical protein	2	Q104H	L66I							
TE32_07715	Transposase (integrase catalytic subunit)	2	E244D	I223N							
tufB	Elongation factor Tu	2	Y201Y	E381E							
tnpA	IS6 family transposase	2	N184D	G184D							
ampC	Beta-lactamase	2	G247S	V236A							
HMPREF0010_03281	Glycosyltransferase	2	F225S	L341S							
ygeV	Transcriptional regulator	2	R284H	A140T							
Hypothetical protein_2	Hypothetical protein	2	I146I	G56E							

*Genes identified as being independently mutated in two or more of the lineages.*

The mutation in *adeRS* appeared four times. *adeRS* was involved in the regulation of efflux pump *adeABC*. The result of MICs for tigecycline showed the mutation in *adeRS* increased the tigecycline resistance in *A. baumannii* (**Table 2**). The result illustrated the evolution steps of tigecycline resistance *in vivo* in *A. baumannii*.

**Table 2:** adeRS mutation conferred tigecycline resistance.

Pair	Strains	TGC(MG/L)	Mutation
1A	XH715	2	
1B	XH717	4	adeR E204A
2A	XH738	2	
2B	XH739	12	adeS Y31N
3A	XH829	2	
3B	XH830	6	adeS R161C
4A	XH833	2	
4B	XH834	12	adeS N127K

## Discussion

In this study, to better understand the genotypic and phenotypic properties underlying the worldwide distribution and evolution of a successful lineage of *A. baumannii*, we explored the evolution pathway of *A. baumannii in vivo*. *A. baumannii* comprises three major international lineages: EC I, II and III (Dijkshoorn et al., 1996; van Dessel et al., 2004). ICL-II, a subgroup of EC II, has spread globally and is the dominant CC of carbapenem-resistant *A. baumannii* (Ruan et al., 2013). Our result are also consistent with those of a recent study by Ruan et al. (2013), who found that isolates from ICL-II exhibited greater resistance to antibiotics than did non-ICL-II isolates. The explanation of the greater resistance might be that isolates from ICL-II harbored more resistance genes than non-ICL-II isolates did. To assess the contribution of mobile elements to the development of antibiotic resistance, we detected the insertion of IS in all of the genome-sequencing isolates to determine the role of IS in the expression of antibiotic resistance genes in *A. baumannii*. IS*Aba1* and IS26 appeared more frequently in ICL-II isolates than in non-ICL-II isolates, which might provide a higher potential to acquire and express resistance gene in the ICL-II isolates.

Two major factors might contribute to the persistence of *A. baumannii* in the host: more resistance and more mucoid. Our data showed that *A. baumannii* demonstrated resistance to major antimicrobial agents. Antibiotic resistance might provide *A. baumannii* with a selective advantage in an environment where bacteria would be exposed to antibiotics (Visca et al., 2011). Furthermore, our data showed that *A. baumannii* became more resistant to CAZ, AZM, and S after within-host evolution. It might cause by ST switching that the former bacteria was replaced by ICL-II strains. Moreover, four isolate pairs presented tigecycline resistance evolution *in vivo*. They harbored mutations in *adeRS*, and the MICs for tigecycline increased (Ruzin et al., 2007). Thus, *A. baumannii* was able to become more resistant to eradication by antibiotics.

A more mucoid phenotype indicated an overproduction of capsular exopolysaccharides, which was caused by the mutations in *ptk* (Geisinger and Isberg, 2015). The overproduction of capsular exopolysaccharides would help bacteria colonize newly available spaces by producing adhesins, and then protect the colonized niche from encroachment by competitors. Especially at the air–liquid interface, the overproduction of capsular exopolysaccharides would push the bacteria themselves into more nutrient and oxygen-rich regions, while smothering their competitors (Hibbing et al., 2010). Our results showed that the overproduction of capsular exopolysaccharides also resulted in higher motility in *A. baumannii* (McQueary et al., 2012). The capsular exopolysaccharides also have demonstrated roles on biofilm modulation (Geisinger and Isberg, 2015). It was proposed that *A. baumannii* would use motility to outcompete other bacteria for more suitable regions *in vivo* and to increase resistance against desiccation and detergents via biofilm. As a consequence, becoming more mucoid would promote the persistence of *A. baumannii* within the host.

Previous studies in *Burkholderia dolosa* and other organisms *have* identified convergent evolution patterns, including decreased virulence and increased antimicrobial resistance (Lieberman et al., 2011). The convergent evolution indicated that bacteria undergo special genetic changes *in vivo*, which helped the bacteria keep a substantial and sustained niche shift away from becoming pathogenic. We observed point mutations, insertions, deletions and copy number dynamics during within-host evolution of *A. baumannii* in this study. The point mutation in *adeRS* led to increased antimicrobial resistance, which confirmed previous findings (Lieberman et al., 2011). We also detected the amino acid changes in AmpC, which were located in the AmpC Ω-loop domain. The amino acid alteration in AmpC might result in the extension of the antibiotic-resistance spectrum (Tian et al., 2011), although the more common mechanism to increase AmpC activity is through the hyper-production of AmpC via inserting ISAba1 into the promoter of the *ampC* gene (Heritier et al., 2006). OmpA is a β-barrel porin in *A. baumannii* (Smith et al., 2007). OmpA played a role in biofilm formation and in interaction with eukaryotic cells (Gaddy et al., 2009). The overexpression of *ompA* was a risk factor associated with pneumonia, bacteremia and mortality in *A. baumannii* (Sanchez-Encinales et al., 2017). OmpA also played a role in antimicrobial resistance in *A. baumannii* (Smani et al., 2014). Bacterial tRNA adenosine deaminase (TadA) would convert adenosine to inosine at the wobble position of tRNA^Arg2^. This process enabled the single tRNA to recognize three different arginine codons in mRNA (Losey et al., 2006). It was shown that *tadA* is an essential gene in *E. coli*, highlighting the importance of inosine at the wobble position in prokaryotes (Wolf et al., 2002). Elongation factor Tuf is a conserved protein acted as a surface exposed plasminogen-binding protein (Kunert et al., 2007). *A. baumannii* EF-Tu was associated with the bacterial cell surface, OMVs and fibronectin (Dallo et al., 2012). EF-Tu would active plasminogen to active plasmin, then active plasmin proteolytically degrade fibrinogen and the key complement component C3b. EF-Tu was considered as multifunctional protein which play a role in virulence of *A. baumannii* via aiding evasion of the complement system (Koenigs et al., 2015). As mentioned above, the ICL-II strains harbored large number of IS*Aba1*. The high activity of IS*Aba1* led to copy number dynamics in OXA-23, which was observed in within-host evolution in this study. It was reported that multiplication of *bla*_OXA-23_ was common in clinical *A. baumannii* (Hua et al., 2016). Another explanation might be the usage of carbapenem in a clinical context. With increased exposure to the selective pressure of carbapenem, the strain with high copy number of OXA-23 might be then selected for. The OXA-23 gene had been mobilized to form Tn2008, Tn2008B, Tn2006, and Tn2009. Tn2006 was most frequently observed worldwide (Nigro and Hall, 2016). It was reported that Tn2008 were detected in 96.4% isolates and Tn2006 in 4.6% isolates in carbapenem-resistant *A. baumannii* in Shanghai, China (Chen et al., 2017). In this study, Tn2006 presented in 37% isolates, while 54% isolates harbored Tn2009. The transposons contributed significantly to the dissemination of OXA-23, although the isolation rate of transposons was different. It was reported that the commonly mutated genes included *pmrAB, adeRS*, transporters, iron acquisition gene, motility and adhesion related genes and the tyrosine kinase *wzc* (*ptk)* gene during *in vivo* evolution in *A. baumannii* (Wright et al., 2016). The gene *adeRS* and *ptk* were shared between Wright et al. (2016) and our study, which demonstrated the existence of mutation hotspot in within-host evolution in *A. baumannii*.

Approximately half of the isolates from the same patients harbored different STs. The significance of this observation is equivocal and may indicate that the latter isolate was a re-infection or that it gained a growth advantage within the host. We could not differentiate between these possibilities for the numbers of isolates from the same patients were limited. The resistance genes in the isolates were predicted by *in silico* profiles using WGS data against the ResFinder database (Zankari, 2014). Only some antibiotic resistance profiles were predicted, which indicated that further experiments are required to fully understand the resistance mechanism in *A. baumannii*, although the resistance database was useful for some predictions.

The strains used in this study were isolated from sputum. This is a limitation of this study that only single isolate was sequenced from each sample, then we were not able to detect the population of the strains that were presented in a patient. To figure out the population of the strains, multiple isolates at multiple isolated time need to be analyzed. Our study focused on single isolate of *A. baumannii* evolved in host. Meanwhile, multiple studies already used isolates from sputum to research *in vivo* evolution. Marvig et al. (2015) sequenced the whole genomes of a total of 474 isolates of *Pseudomonas aeruginosa* isolated from sputum. Wright et al. (2016) detected the genome dynamics of MDR *A. baumannii* during infection and treatment. The 54% (74/136) strains were isolated from sputum in Wright et al. (2016) study. Overall, this study explored the within-host evolution in *A. baumannii* in real time and highlighted the ability of NGS to explore evolutionary events within resistant organisms.

## Nucleotide Sequence Accession Numbers

The whole-genome shotgun projects of *A. baumannii* strains have been deposited at DDBJ/EMBL/GenBank under the accession numbers LYEX00000000- LYLM00000000, respectively. The versions described in this paper are versions LYEX00000000- LYLM00000000.

## Author Contributions

XH analyzed the genomic data and drafted the manuscript. ZZ, QY, QS, QX, JW, KS, FZ, LS, ZR, and YJ performed the experiment. XH and YY designed the study and drafted the manuscript. All authors read and approved the final manuscript.

## Conflict of Interest Statement

The authors declare that the research was conducted in the absence of any commercial or financial relationships that could be construed as a potential conflict of interest.

## Footnotes

1 https://github.com/katholt/srst2

## Supplementary Material

FIGURE S1 The overview of the 172 genome-sequenced *A. baumannii* isolates. **(A)** Each symbol represents a genome-sequenced *A. baumannii* isolate. The *y*-axis indicates the patient from whom the isolate was obtained, while *x*-axis indicates the sampling time for each isolate. Colors are indicated for isolates belonging to different MLST patterns. The line connecting two symbols indicated the time between two isolate samplings from the same patient. **(B)** The distribution of the isolation range for all 172 genome-sequenced isolates. Colors are indicated for isolates belonging to different MLST patterns.

FIGURE S2 Resistance genes detected in these genome-sequencing isolates of *A. baumannii*.

FIGURE S3 Distribution of SNPs in Group B compared with Group A in *A. baumannii*. **(B)** Pathoadaptive genes mutated in Group B compare with Group A. The blue squares in the large matrix denote whether the genes underwent non-synonymous mutation. The analysis was performed based only on non-synonymous mutated genes that were altered independently in two of the lineages.

FIGURE S4 Schematic of the ptk domains indicates the location of point mutations in the conserved motifs. White: conserved motif.
