# Supplementary figures and images for: Evolution of Acinetobacter baumannii In Vivo: International Clone II, More Resistance to Ceftazidime, Mutation in ptk
Source: Front Microbiol. 2017 Jul 10;8:1256. doi: 10.3389/fmicb.2017.01256 (PMC5502287; doi:10.3389/fmicb.2017.01256)

A

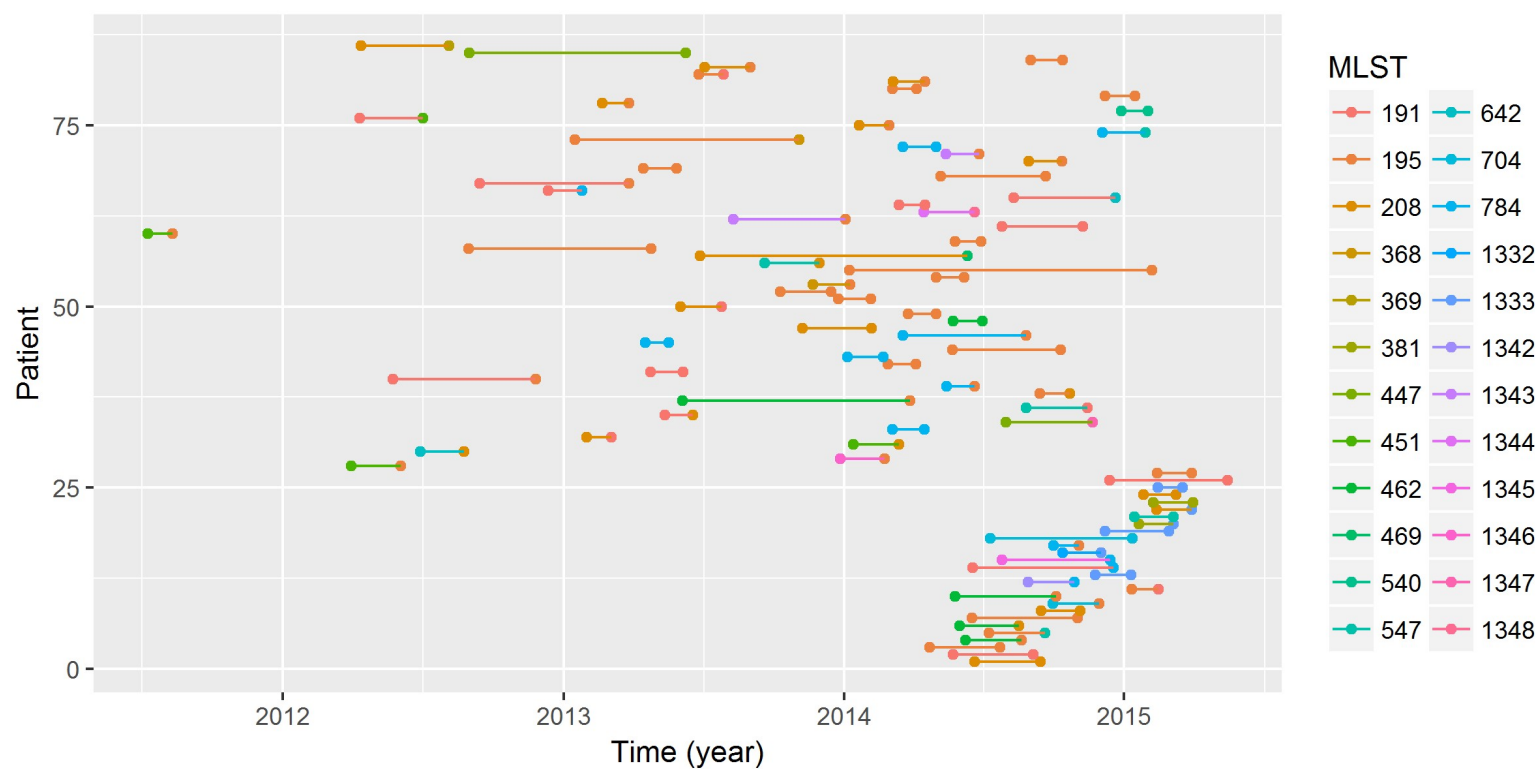

B

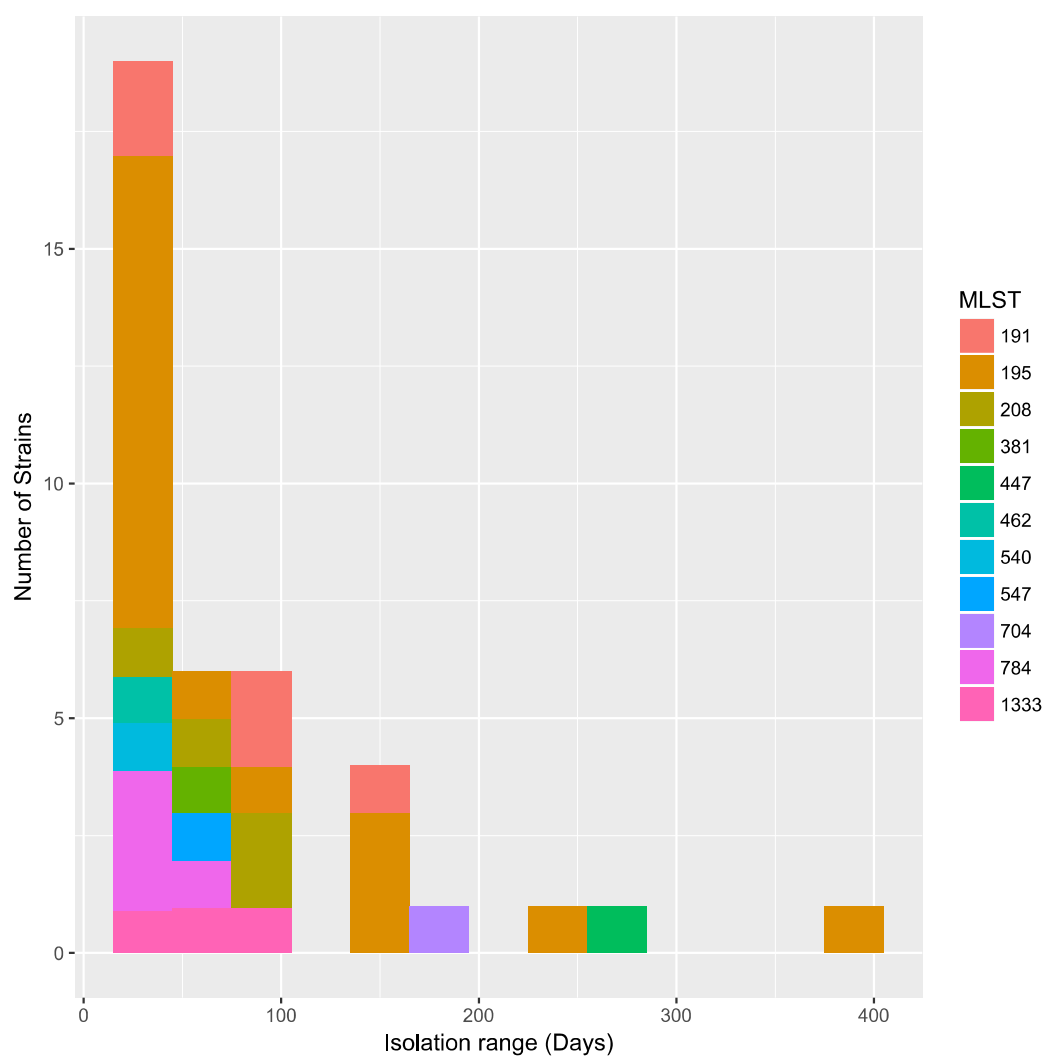

Supplement: FIGURE S1 — The overview of the 172 genome-sequenced A. baumannii isolates. (A) Each symbol represents a genome-sequenced A. baumannii isolate. The y-axis indicates the patient from whom the isolate was obtained, while x-axis indicates the sampling time for each isolate. Colors are indicated for isolates belonging to different MLST patterns. The line connecting two symbols indicated the time between two isolate samplings from the same patient. (B) The distribution of the isolation range for all 172 genome-sequenced isolates. Colors are indicated for isolates belonging to different MLST patterns. [file Image_1.PDF]

A

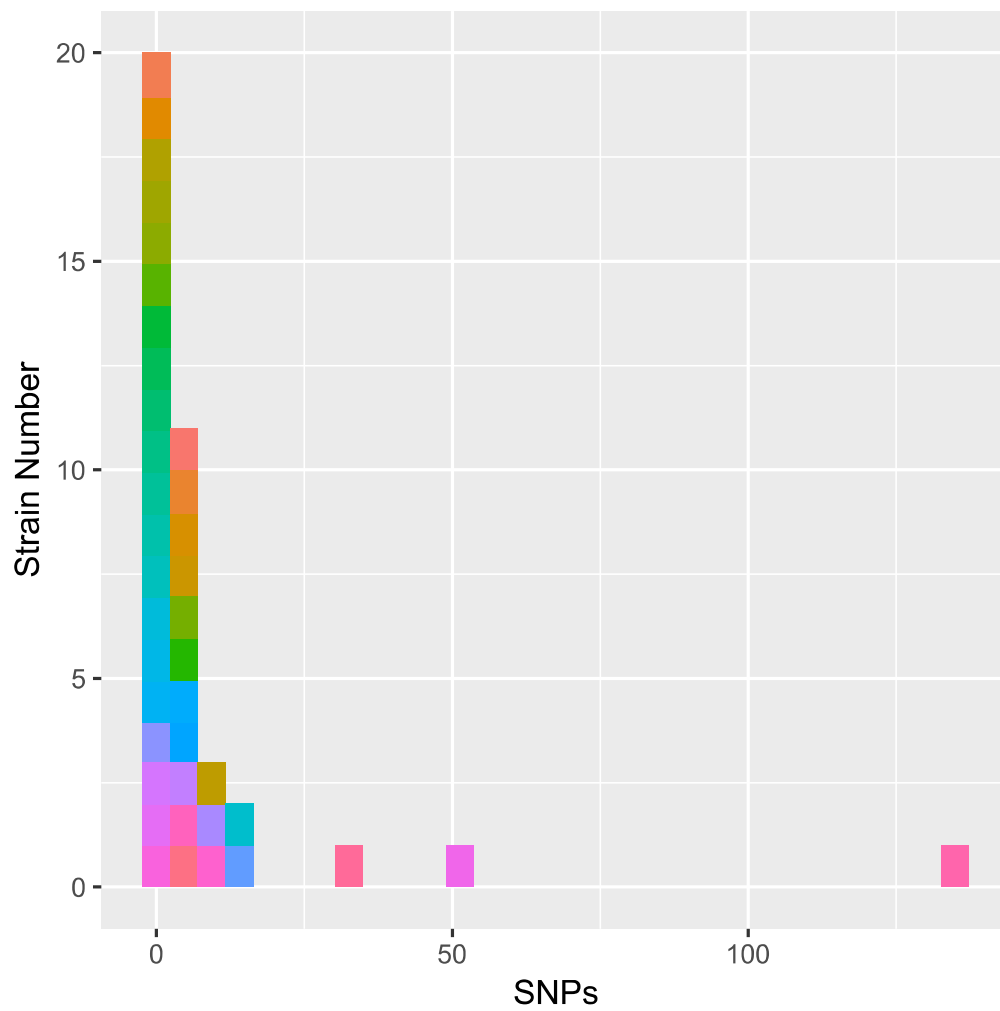

B

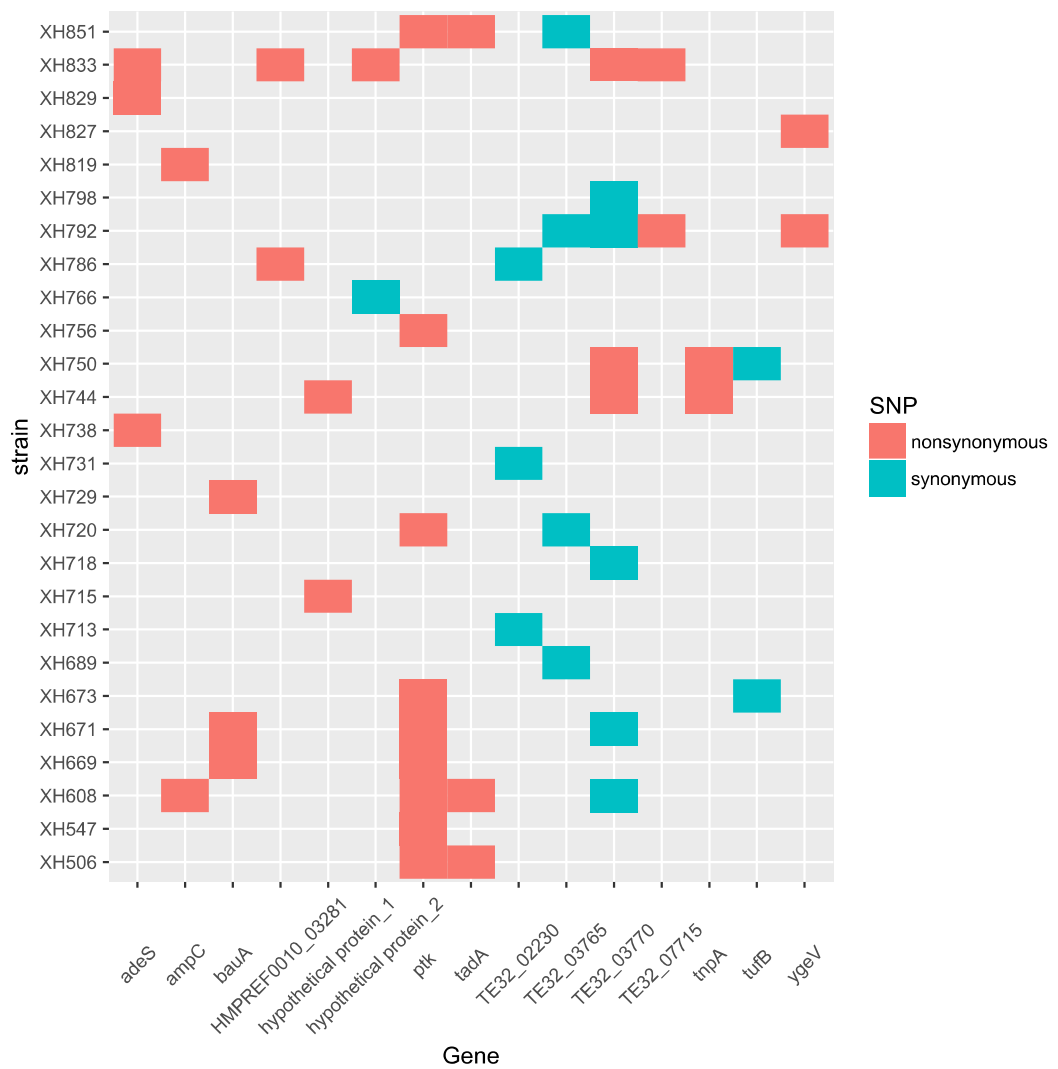

Supplement: FIGURE S3 — Distribution of SNPs in Group B compared with Group A in A. baumannii. (B) Pathoadaptive genes mutated in Group B compare with Group A. The blue squares in the large matrix denote whether the genes underwent non-synonymous mutation. The analysis was performed based only on non-synonymous mutated genes that were altered independently in two of the lineages. [file Image_3.PDF]

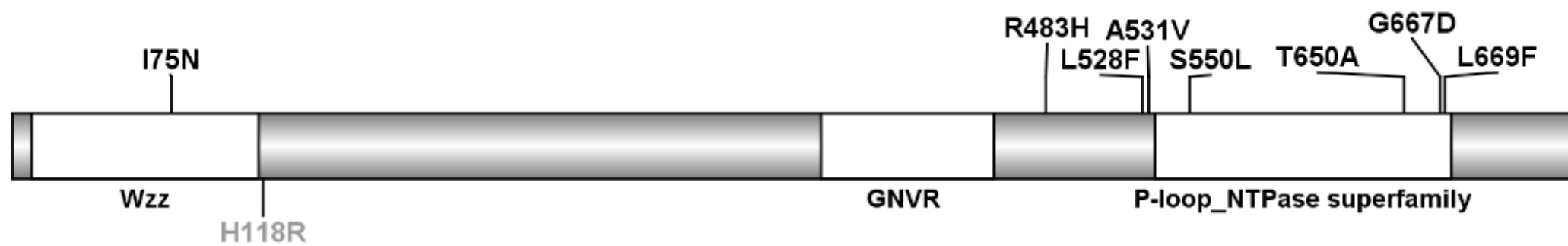

Supplement: FIGURE S4 — Schematic of the ptk domains indicates the location of point mutations in the conserved motifs. White: conserved motif. [file Image_4.PDF]
